# Are topical insect repellents effective against malaria in endemic populations? A systematic review and meta-analysis

**DOI:** 10.1186/1475-2875-13-446

**Published:** 2014-11-21

**Authors:** Anne L Wilson, Vanessa Chen-Hussey, James G Logan, Steve W Lindsay

**Affiliations:** School of Biological and Biomedical Sciences, Durham University, South Road, Durham, DH1 3LE UK; London School of Hygiene and Tropical Medicine, Keppel Street, London, WC1E 7HT UK

## Abstract

**Background:**

Recommended vector control tools against malaria, such as long-lasting insecticidal nets (LLINs) and indoor residual spraying (IRS), mainly target mosquitoes that rest and feed on human hosts indoors. However, in some malaria-endemic areas, such as Southeast Asia and South America, malaria vectors primarily bite outdoors meaning that LLINs and IRS may be less effective. In these situations the use of topical insect repellents may reduce outdoor biting and morbidity from malaria. A systematic review and meta-analysis was conducted to assess the efficacy of topical insect repellents against malaria.

**Methods:**

Studies were identified using database searches (MEDLINE, EMBASE, Web of Science and clinical trials registers), as well as reference list searches and contact with researchers. Randomized and non-randomized controlled trials were included that assessed the effect of topical repellents (all active ingredients and concentrations) on *Plasmodium falciparum* or *Plasmodium vivax* malaria or infection in malaria-endemic populations. Meta-analysis of clinical data was conducted in order to generate summary risk ratios.

**Results:**

Ten trials met the inclusion criteria. Studies varied in terms of repellent active ingredient and formulation, co-interventions, study population, compliance, and follow-up period. Topical repellents showed an 18% protective efficacy against *P. falciparum* malaria, although this was not significant (95% CI: -8%, 38%). Similarly, the average protective efficacy of topical repellents against *P. vivax* malaria did not reach significance (protective efficacy: 20%, 95% CI: -37%, 53%). Exclusion of non-randomized trials from the meta-analysis did not alter the findings.

**Conclusions:**

Although topical repellents can provide individual protection against mosquitoes, the results of this meta-analysis indicate that topical repellents are unlikely to provide effective protection against malaria. However, there was substantial heterogeneity between studies included and the relatively small number of studies meant that this heterogeneity could not be fully explored in the analysis. Further well-designed trials of topical repellents at appropriate doses and alternative modes of repellent delivery, such as spatial repellents and long-lasting insecticide-treated clothing, are required.

**Electronic supplementary material:**

The online version of this article (doi:10.1186/1475-2875-13-446) contains supplementary material, which is available to authorized users.

## Background

Malaria is a major cause of morbidity and mortality in developing countries. In 2012, the World Health Organization (WHO) estimated that there were 207 million cases of malaria, which caused approximately 627,000 malaria deaths [1]. The parasites that cause malaria, primarily *Plasmodium falciparum* and *Plasmodium vivax*, are transmitted by the bite of female mosquitoes belonging to the genus *Anopheles*. Vector control plays a major part in malaria control and recommended vector control tools include long-lasting insecticidal nets (LLINs) and indoor residual spraying (IRS). Both tools have contributed to the large declines in malaria observed over the past decade. It is therefore of great concern that insecticide resistance in malaria vectors is widespread in sub-Saharan Africa (SSA), particularly to pyrethroids, the only insecticide class suitable for impregnation of LLINs [2].

Both LLINs and IRS aim to control malaria vectors that feed on human hosts and rest indoors. However, in many malaria-endemic areas, including Southeast Asia and South America, biting occurs mainly outdoors. For example, the most important malaria vectors in the Greater Mekong Subregion in Southeast Asia are *Anopheles dirus*, *Anopheles minimus* and *Anopheles maculatus* which often bite outdoors and prior to 22.00 hours before people who own LLINs are protected by them [3, 4]. Scale up of LLINs in SSA has been associated with a change in vector dominance from the predominantly indoor biting vector *Anopheles gambiae s.s*. to the outdoor biting vector *Anopheles arabiensis* [5–7]. There is also evidence of behavioural resistance of malaria vectors in response to the wide-scale use of IRS and LLINs [8]. Malaria vectors may be adapting their behaviour to early evening and dawn biting in response to reduced availability of blood meals at night when people are sleeping under LLINs. Indeed, studies in SSA [6, 9] and the Pacific [10, 11] have reported an increase in early evening biting of malaria vectors following roll-out of LLINs or IRS. Increasing development of urban areas and availability of electricity means that people are staying awake for longer and are exposed to outdoor-biting mosquitoes in the evening [12]. In addition, some populations groups, for example hunters, rubber tappers or forest workers that are active at night or sleep in the forest [13, 14] are at high risk of malaria transmission from outdoor-biting mosquitoes. Based on this information, there is a need for vector control tools to protect people against outdoor-biting vectors.

Topical insect repellents protect users from mosquito bites as people go about their daily activities and therefore offer a potential tool against outdoor-biting mosquitoes. It is likely that people have been using repellents to prevent insect bites since prehistory [15]. Early repellents were largely plant derived and include some repellents that are still in use today, such as citronella (oil derived from plants of the *Cymbopogon* genus), neem (leaves from *Azidarachta indica*) and lemon eucalyptus (*Eucalyptus maculata citriodon*). *N*,*N*-diethyl-m-toluamide (DEET), developed in the 1950s, is the most effective repellent available [16]. Topical insect repellents are very successful at reducing outdoor biting at any time of the day from a wide range of insects, but this protection is short-lived. For example, the current ‘gold standard’ repellent, DEET, applied topically will provide approximately six hours of protection under field conditions, although this is dependent on the formulation [17, 18].

A number of trials of topical repellents against malaria have been conducted but it is necessary to synthesize the results of these trials in order to inform policy decisions on use of topical repellents. Narrative and systematic reviews of topical insect repellents for personal protection have been conducted but these did not use meta-analysis [19–21]. Therefore, a systematic review and meta-analysis of randomized and non-randomized controlled trials was conducted to determine the efficacy of topical insect repellents against *P. falciparum* and *P. vivax* malaria or infection in malaria-endemic populations.

## Methods

### Literature search

Recommendations made by the Preferred Reporting Items for Systematic Reviews and Meta-Analyses (PRISMA) group were followed where possible [22, 23] (PRISMA Checklist: Additional file 1).

A systematic search of the literature was performed in January 2014 and updated in July 2014. Medline (1946-), Embase (1980-) and Web of Science databases were searched using search terms including ‘malaria’ and ‘insect repellents’ and using MeSH terms where appropriate. No language restrictions were placed on this search. More detail on the search strategy is given in Additional file 2. In addition, clinical trials databases [24, 25] were searched, reference lists of identified manuscripts were checked and researchers were contacted to identify ongoing studies.

ALW screened the abstracts of the citations identified for potentially relevant studies and full text documents were obtained for those publications deemed to be relevant. The articles were scrutinized to ensure that multiple publications from the same study were included only once.

### Study inclusion and exclusion criteria

Studies identified were assessed against inclusion and exclusion criteria by ALW and SWL. Randomized and non-randomized controlled trials of topical repellents in endemic populations were included. Trial interventions included any topical insect repellent, regardless of active ingredient or concentration used. Studies including co-interventions (usually insecticide-treated nets (ITNs) or LLINs) were included. Control arms received either no repellent, placebo repellent or co-interventions. Studies were included if they assessed the efficacy of topical repellents against either *P. falciparum* or *P. vivax* malaria cases or infection (self-reported or diagnostically confirmed using microscopy or a rapid diagnostic test). Both incidence and prevalence measures were included.

Studies in travellers to malaria-endemic regions were excluded since the susceptibility of these populations to malaria and other factors, such as trial duration and compliance, would likely differ. Studies of insect repellent impregnated clothing and spatial repellents were also excluded. Studies assessing only entomological outcomes and arm-in-cage/laboratory studies/semi-field studies were excluded since the focus was primarily on determining whether repellents impacted on malaria morbidity.

### Data extraction and analysis

ALW and VC-H independently extracted data from included studies into a standardized form capturing data on trial location, study population, randomization, blinding methods, repellent formulation, estimated coverage or compliance and method of estimation, type of control, co-interventions, outcome measures, and length of follow-up from each trial. If these were not presented in the report, the trial location was used to find malaria endemicity, *Plasmodium* species and *Anopheles* vectors present. Where the *Plasmodium* species was not determined, the protective efficacy was attributed to the most common *Plasmodium* species which was identified either from the manuscript or expert opinion.

Clinical outcomes were reported as either risks, odds or rates of disease or infection in the published papers. For consistency across studies, risks of disease or infection were used in the meta-analysis. In the few cases where studies reported rates, risks were calculated using data on the number of cases and size of the study populations which was included in the published papers. The meta-analysis was conducted using unadjusted data. This decision was taken due to the small number of trials identified that reported adjusted effect estimates and the inconsistency across measures reported (adjusted rate and odds ratios). The metan command was used to perform meta-analysis in Stata 13 (StataCorp, Texas, USA). Due to the higher risk of bias in studies that were non-randomized, the meta-analysis was conducted both including and excluding these studies. Statistical heterogeneity was assessed using a χ^2^ test. Due to the small number of studies in each comparison, the data were said to be heterogeneous if the χ^2^ test p value was less than or equal to 0.1 [26]. The I^2^ statistic was used to quantify the degree of heterogeneity. I^2^ was calculated as I^2^ = ((Q - d.f.)/Q) x 100%, where Q is the χ^2^ statistic and d.f. is the number of degrees of freedom. Due to the high levels of statistical heterogeneity found and the *a priori* assessment that the studies were indeed heterogeneous (different repellent types, study sites, etc.), the summary effect measure was calculated using random effect meta-analysis, rather than fixed effect meta-analysis. Protective efficacy (PE) was calculated as PE =1- (risk ratio of clinical disease or infection during the intervention period) × 100%. PE (or relative risk reduction) can be interpreted as the percentage reduction in risk of clinical disease or infection associated with the intervention. A standard formula was used to calculate 95% confidence intervals for risk ratios [27].

### Risk of bias assessment

ALW assessed the risk of bias in the studies using the Effective Practice and Organization of Care (EPOC) risk of bias assessment form [28]. Risk of bias for each of the domains was graded as low, high or unclear risk.

## Results

### Study selection

**Figure 1:**
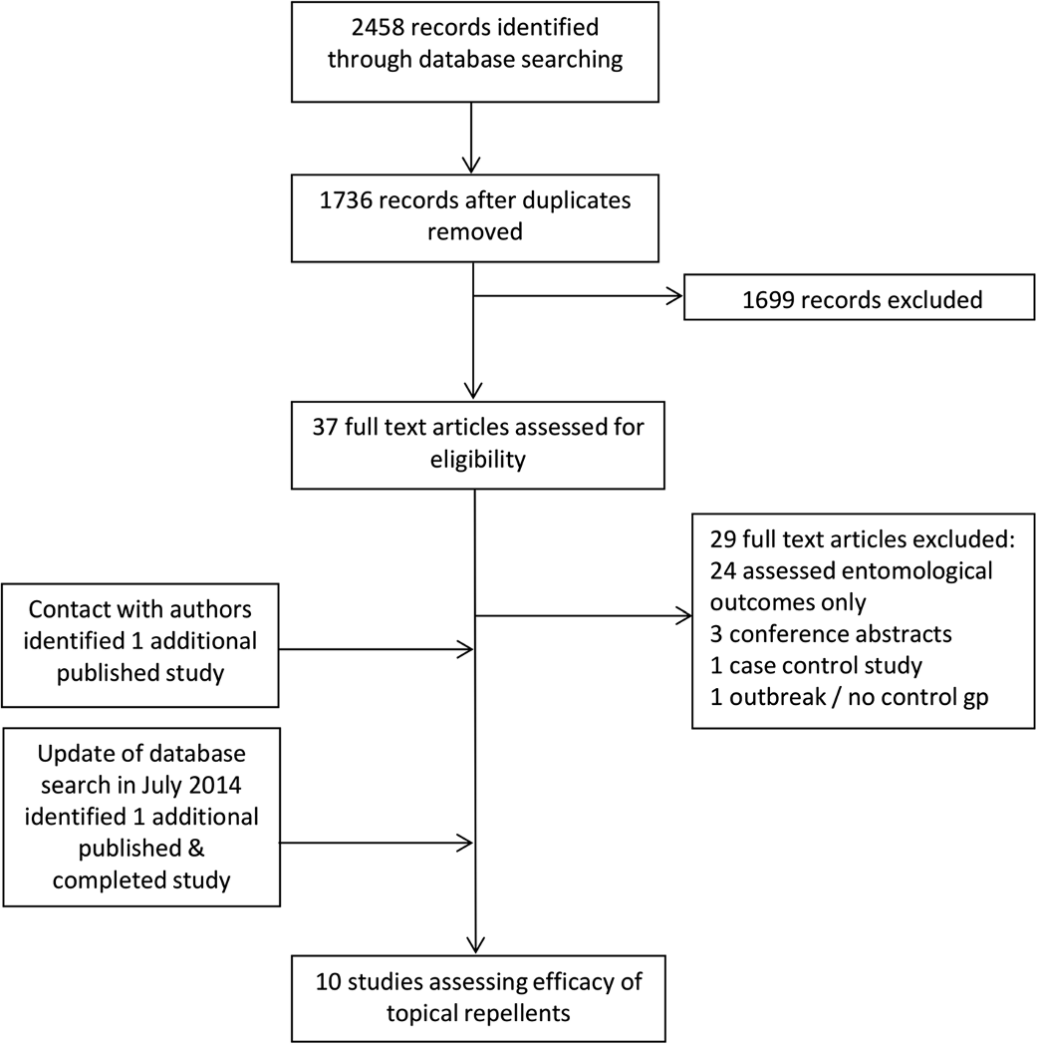
**Flow chart of study selection (adapted from** [22] **).**

The initial systematic literature search identified 1,736 unique records (Figure 1). 1,699 records were excluded based on review of the title and abstract. The majority of these studies related to use of insecticide treated materials (e.g. LLINs) or chemoprophylaxis in travellers, or described risk factors for malaria. 37 full text records were reviewed and of these eight studies met the inclusion/exclusion criteria. Contact with experts identified one additional study [12]. The update of the search in July 2014 identified two additional studies – one of which was published [29]. The second study was identified from Clinicaltrials.gov [24] (Identifier: NCT01663831) and could not be included because the data were still being analysed. Therefore, the total number of studies included in the review was ten. One of these studies was available as a study report [12] but was later published as a peer-reviewed manuscript [30]. Figures from that manuscript were used for the analysis.

### Study characteristics and risk of bias

The ten studies identified were conducted in Africa (Ethiopia, Ghana, Tanzania), Asia (India, Lao People’s Democratic Republic (PDR), Pakistan, Thailand) and South America (Bolivia and Ecuador and Peru) (Additional file 3). Three studies assessed the effect of repellents on *P. falciparum* malaria/infection [30–32], five studies assessed the effect on both *P. falciparum* and *P. vivax* malaria/infection [29, 33–36] and two studies did not determine the *Plasmodium* species [37, 38]. Four studies measured malaria incidence [30, 32, 37, 38], four studies measured incidence of infection [33–36] and two measured parasite prevalence [29, 31]. Studies utilized a range of topical insect repellents, the most common being DEET and four of the ten studies used ITNs or LLINs as a co-intervention. The study characteristics of these trials are summarized in Additional file 3.

Risk of bias assessment found that studies were generally at low risk of bias, although poor description in the published papers meant that many parameters could only be classified as ‘unclear’ (Additional file 4). Seven studies were reported as being randomized trials (although the randomization process was not well described in several papers), and it was assumed that three trials making no mention of randomization were non-randomized [31, 32, 38]. In one of these studies by Vittal *et al*., baseline malaria incidence was significantly lower in the intervention group compared to control group [38], and in another by Dadzie *et al*. baseline malaria prevalence was significantly greater in the intervention village at baseline, although this would most likely serve to bias the effect size downwards [31]. The study in Tanzania reported that socio-economic status was higher in the control arm, suggesting that randomization was imbalanced [30]. Only one study identified [37] did not use diagnostic confirmation of malaria, instead relying on self-reporting which the researchers ‘validated’. This study reported that agreement between self- and professional-diagnosis (including diagnostic confirmation) was 80-90%.

### Results of individual studies

Of the nine studies that assessed the efficacy of topical repellents against *P. falciparum* malaria, only one of these by Rowland *et al*. reported a significant protective efficacy [35]. Only one of the seven studies that assessed the efficacy of topical repellents against *P. vivax* malaria reported a significant protective efficacy [33]. Individual study results are reported in Tables 1 and 2 and further detail is given in Additional file 5.

**Table 1:** **Efficacy of topical repellents against** ***Plasmodium falciparum***

Study	Repellent		Control		Risk ratio (95% confidence intervals)
	Cases	Population at risk	Cases	Population at risk	
Chen-Hussey *et al*. [34]	35	3,947	33	3,961	1.06 (0. 66-1.71)
Dadzie *et al*. [31]	54	205	47	204	1.14 (0.81-1.61)
Deressa *et al*.^1^ [29]	23	2,399	19	2,273	1.15 (0.63 - 2.10)
Dutta *et al*. [32]	-	-	-		Yr 1: 1.16 (0.85-1.58)
					Yr 2: 1.20 (0.83-1.72)
Hill *et al*. [33]	1	2,041	6	1,967	0.16 (0.02-1.33)
Kroeger *et al*.^2^ [37]	8.5%		6.7%		1.27^3^
McGready *et al*.^4^ [36]	40	379	30	202	0.71 (0.46-1.11)
Sangoro *et al*.^5^ [30]	115	2,224	137	2,202	0.83 (0.65-1.06)
Rowland *et al*. [35]	23	618	47	530	0.42 (0.26-0.68)

^1^Denominator is average of two follow up surveys, number of infections is combined total from two follow-up surveys - based on assumption that infections at 2-month time point were new infections (1 month between follow-up surveys); ^2^Trial conducted in two sites. This data is from Ecuador where according to manuscript 86% of cases were usually due to *P. falciparum*. Since parasite species of cases was not determined, these cases were attributing to *P. falciparum*; ^3^Counts and denominators not reported in manuscript so unable to calculate 95% confidence intervals; ^4^Cases and denominator back-calculated from percentages and confidence intervals reported in paper; ^5^number of cases/denominator taken from published manuscript not study report.

**Table 2:** **Efficacy of topical repellents against** ***Plasmodium vivax***

Study	Repellent		Control		Risk ratio (95% confidence intervals)
	Cases	Population at risk	Cases	Population at risk	
Chen-Hussey *et al*. [34]	14	3,947	16	3,961	0.88 (0.43-1.80)
Deressa et al. ^1^ [29]	21	2,399	17	2,273	1.17 (0.62 - 2.21)
Hill *et al*. [33]	14	2,041	66	1,967	0.20 (0.12-0.36)
Kroeger *et al*. ^2^ [37]	17.9%		24.1%		0.74^3^
McGready *et al*. ^4^ [36]	67	316	70	266	0.81 (0.60-1.08)
Rowland *et al*. [35]	103	618	62	530	1.42 (1.06-1.91)
Vittal *et al*. ^5^ [38]	8	228	13	411	1.11 (0.47-2.64)

^1^Denominator is average of two follow up surveys, number of infections is combined total from two follow-up surveys - based on assumption that infections at 2-month time point were new infections (1 month between follow-up surveys); ^2^Trial conducted in two sites. This data is from Peru where according to manuscript 86% of cases were usually due to *P. vivax*. Since parasite species of cases was not determined, these cases were attributing to *P. vivax*; ^3^Counts and denominators not reported in manuscript so unable to calculate 95% confidence intervals; ^4^Cases and denominator back-calculated from percentages and confidence intervals reported in paper; ^5^Number of cases is combined total from two years of follow up.

### Synthesis of results

Two studies could not be included in the meta-analysis. The trial conducted by Kroeger *et al*. in Ecuador and Peru did not report numbers of cases or denominators [37]. This was also the only study included which relied on self-reported malaria incidence. Dutta *et al*. seemed to misinterpret the results of their study in the published paper stating that risk ratios greater than 1 were protective [32]. Attempts to contact the authors to clarify and obtain the study data were unsuccessful and so this study was excluded from the meta-analysis.

**Figure 2:**
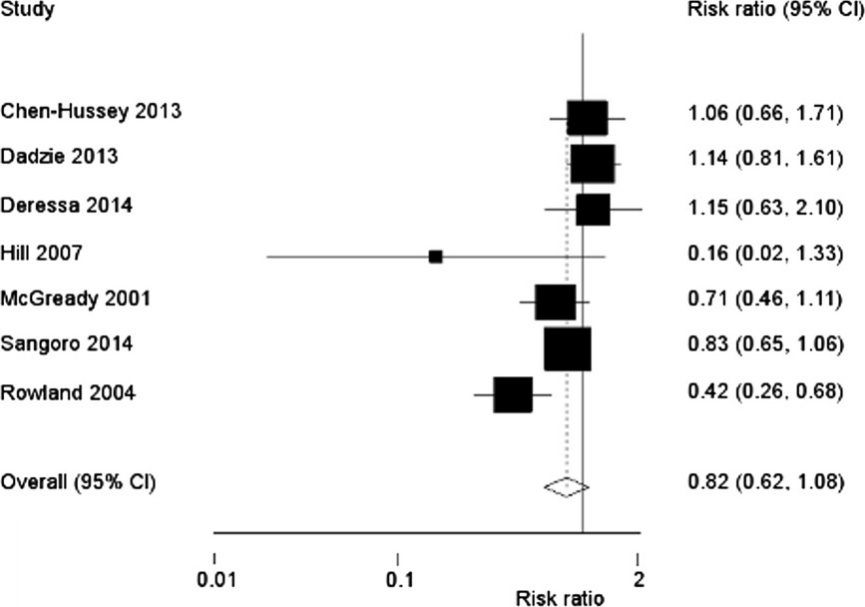
**Forest plot showing risk ratios and summary effect estimate of topical insect repellent against** ***Plasmodium falciparum*** **malaria (random effects meta-analysis).**

**Figure 3:**
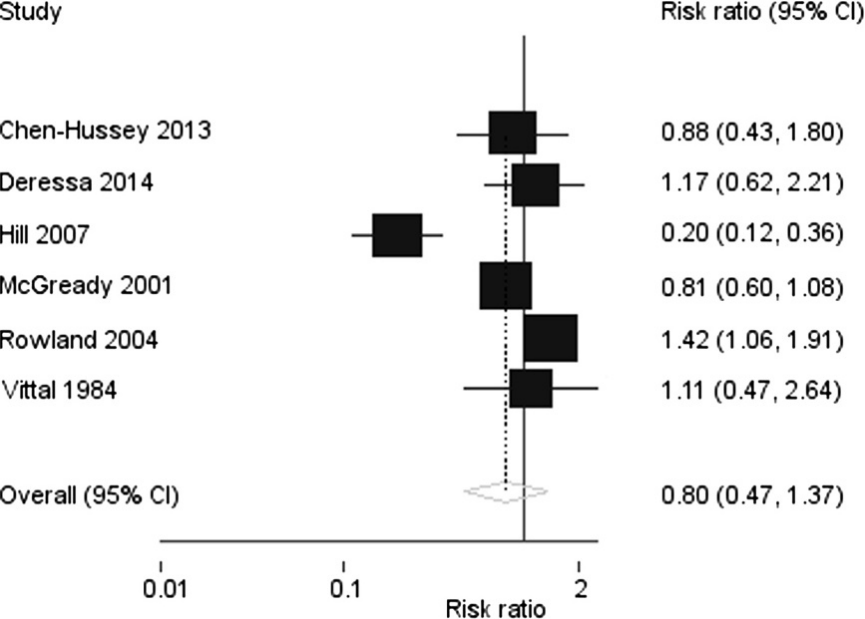
**Forest plot showing risk ratios and summary effect estimate of topical insect repellent against** ***Plasmodium vivax*** **malaria (random effects meta-analysis).**

The combined summary risk ratio for the effect of topical repellents on *P. falciparum* malaria or infection was 0.82 (95% CI: 0.62, 1.08, p = 0.2) (Figure 2). There was substantial heterogeneity across studies (χ ^2^ p value = 0.01, I^2^ = 62%). Similarly the protective efficacy of topical repellents against *P. vivax* malaria or infection was not significant (risk ratio: 0.80 (95%CI: 0.47, 1.37, p = 0.4) (Figure 3). There was considerable heterogeneity across studies (χ^2^ p value <0.001, I^2^ = 87%). When non-randomized trials were excluded from the meta-analysis, the risk ratios did not change substantially (*P. falciparum* risk ratio: 0.76, 95%CI: 0.55, 1.03, p = 0.08, *P. vivax* risk ratio: 0.76, 95%CI: 0.42, 1.39, p = 0.4).

## Discussion

This meta-analysis did not show a significant protective effect of topical repellents against either *P. falciparum* (18%, 95% CI: -8%, 38%) or *P. vivax* malaria or infection (20%, 95% CI: -37%, 53%). Calculating the summary effect measure excluding non-randomized trials, did not alter the conclusion – no significant protective effect of topical repellents was observed.

Heterogeneity was high in the meta-analysis indicating substantial variance between the studies. Sources of heterogeneity included varying background rates of malaria, outcome measures (malaria cases or infection), follow-up periods, characteristics of participants (e.g., age), active ingredients, concentration and formulation of the repellent, user compliance, and co-interventions. Due to the small number of studies identified it was not possible to conduct subgroup analysis to account for some of these important differences between studies. The most obvious difference was in study location, which would lead to varying background malaria rates. The interventions also varied; DEET, permethrin and *p*-Menthane-3,8-diol (PMD) were all used at different concentrations and formulations. Compliance varied greatly between studies from 58% in Lao PDR to 98% in Bolivia.

There is strong evidence from a large number of studies that topical repellents protect from mosquito bites [39–44]. Studies included in the review also demonstrated high protection of the repellents against mosquito bites. For example, Moore *et al*. reported a high level of protection from *An. gambiae s.l*. biting in a field trial using human-landing catches in Tanzania [12] and Dadzie *et al*. reported that the biting pressure of *Anopheles* on unprotected individuals averaged 86 bites/man/night, which was significantly reduced to nine bites/person/night among collectors using the NO MAS repellent [31]. However, the results of this meta-analysis suggest that protection from biting in controlled entomological studies does not translate into protective efficacy against clinical malaria. There are a number of potential reasons for this that are discussed briefly here. Firstly, compliance with repellent use may be suboptimal and vary amongst the study population. A mathematical model developed by Kiszewskia and Darling indicates that the probability of avoiding infections is highly sensitive to small changes in compliance and product efficacy – both of which are exponential parameters in the model [45]. In a study setting, compliance is difficult to measure as direct observation is only practicable in a small number of participants. Most of the trials used a combination of self-reported data confirmed by a small number of direct observations. Self-reported data may be unreliable due to courtesy bias whereby participants report using repellent even though they have not used it. It is also difficult to standardize repellent use given that participants may use varying amounts of the lotion each time they apply it leading to varying repellent effects. Secondly, the duration of protection from biting provided by repellents is relatively short. Even though participants may apply the lotion correctly in early evening, waning of the effect of the repellent may mean that participants are unprotected during the night and early morning. The risk of malaria may be even greater if the participant perceives they are protected and so does not comply with use of personal protective measures, such as LLINs. Thirdly, in some of the studies LLINs were used as a co-intervention – indeed, it is unethical to deny LLINs from control groups since they are considered ‘standard best practice’. However, this means that the study needs to show an effect of repellents on top of LLINs, an already highly effective intervention. This poses a problem of ‘statistical power, and the law of diminishing returns’ as noted by Lines and Kleinschmidt [46], whereby large sample sizes are required to have sufficient power to show a small increase in protection on top of LLINs. Lack of power may have been a problem in some of the studies. For example, in Thailand [36] and Tanzania [12, 30] reductions in malaria rates were recorded in repellent users, but the lower than expected overall malaria rates meant that sample sizes were too low for this reduction to reach significance.

Compliance with preventive measures such as topical repellents is dependent on a number of factors including acceptability of the product and biting nuisance. Ensuring high compliance with repellent use is critical in order to prevent diversion of malaria vectors to non-repellent-using individuals, especially if the vector species are strongly anthropophilic. A study in Tanzania showed that placebo users living in a village where 80% of the households used 15% DEET had over four times more mosquitoes resting in their dwellings in comparison to households in a village where nobody used repellent [47]. Some of the better designed studies included in this review attempted to reduce this diversion effect by enrolling a relatively small proportion of the population from villages/camps [12, 34, 35], but this was not the case with all studies or was not described in the papers.

This review assessed the efficacy of topical insect repellents against malaria in endemic populations but did not look at their efficacy when used by travellers. Malaria risk (due to for example immunity or living accommodation) and repellent use is likely to be different in endemic populations and travellers and so the data cannot be extrapolated between these two populations. Since topical repellents are able to reduce biting rates when used correctly [42], it is recommended that travellers continue to use them [20, 48, 49].

This review has a number of limitations which should be noted. Firstly, despite a comprehensive literature search of several databases, clinical trials registers and contact with researchers there is a possibility of missing some relevant studies. However, although a systematic search of grey literature databases was not conducted it is likely that all relevant studies were identified. While ten studies might be considered modest in order to make conclusions on a vector control tool, this is comparable to other systematic reviews of vector control tools (Cochrane reviews on ITNs =22 studies [50], IRS = six studies [51], larvivorous fish =12 studies [52], larval source management =13 studies [53]). Studies were generally at low risk of bias, although many bias parameters could only be rated as ‘unclear’ given the poor reporting in the published studies. Efforts should be made to improve reporting of vector control studies.

## Conclusions

Although entomological evidence is available that topical repellents protect individuals from mosquito bites, the results of this meta-analysis suggest they are ineffective at preventing malaria morbidity. However, there was substantial heterogeneity between studies and the relatively small number of studies identified meant that the effect of this heterogeneity on the summary effect estimate could not be assessed. Therefore it is recommended that further well-designed trials of topical repellents at appropriate doses be conducted. Additionally, research should focus on alternative modes of repellent delivery such as spatial repellents and long-lasting insecticide-treated clothing, which rely less on compliance. Although repellents do not seem to be effective against malaria, they may be effective against other diseases vectored by insects, including dengue and leishmaniasis [54]. Studies of topical repellents against other vector-borne diseases should therefore be conducted.

## Electronic supplementary material

Additional file 1: **PRISMA checklist.** (DOCX 20 KB)

Additional file 2: **Database search terms.** (DOCX 14 KB)

Additional file 3: **Characteristics of included studies.** (DOCX 34 KB)

Additional file 4: **Risk of bias assessment.** (DOCX 28 KB)

Additional file 5: **Detailed efficacy results.** (XLSX 17 KB)

## Abbreviations

DEET: *N*,*N*-diethyl-m-toluamide
EPOC: Effective practice and organization of care
IRS: Indoor residual spraying
ITN: Insecticide-treated net
LLIN: Long-lasting insecticidal net
PDR: People’s Democratic Republic
PE: Protective efficacy
PMD: *p*-Menthane-3,8-diol
PRISMA: Preferred reporting items for systematic reviews and meta-analyses
SSA: Sub-Saharan Africa
WHO: World Health Organization.
