# Supplementary material for: Are topical insect repellents effective against malaria in endemic populations? A systematic review and meta-analysis
Source: Malar J. 2014 Nov 21;13:446. doi: 10.1186/1475-2875-13-446 (PMC4246562; doi:10.1186/1475-2875-13-446)
Supplement: Supplementary file 2 — Additional file 2: Database search terms. (DOCX 14 KB) [file 12936_2014_3597_MOESM2_ESM.docx]

**Additional File 2: Database search terms**

| **Date of search** | **Database** | **Search terms** |
| --- | --- | --- |
| 17^th^ Jan 2014  28^th^ July 2014 | Medline | exp Malaria/  AND  [exp Insect Repellents/ DEET/ "Insect Bites and Stings"/ repellent.mp. / Permethrin/] |
|  | Embase | exp malaria/  AND  [permethrin/ or diethyltoluamide/ or exp insect repellent/ or insect bite/ or repellent.mp.] |
|  | Web of Science | TOPIC: (malaria) AND TOPIC: (repellent) |
