# Supplementary material for: Are topical insect repellents effective against malaria in endemic populations? A systematic review and meta-analysis
Source: Malar J. 2014 Nov 21;13:446. doi: 10.1186/1475-2875-13-446 (PMC4246562; doi:10.1186/1475-2875-13-446)
Supplement: Supplementary file 3 — Additional file 3: Characteristics of included studies. (DOCX 34 KB) [file 12936_2014_3597_MOESM3_ESM.docx]

**Additional file 3 Characteristics of ten unique, eligible studies identified via systematic review**

| **Study** | **Study type** | **Year study conducted** | **Location** | **Study population** | **Intervention** | **Compliance** | **Control** | **Outcomes** | **Measurement** |
| --- | --- | --- | --- | --- | --- | --- | --- | --- | --- |
| Chen-Hussey *et al*. [[1](#_ENREF_1)] | Double blind, household randomized, placebo-controlled trial | 2009-10 | Lao PDR | 1,597 households recruited from agricultural communities. Up to 25% of households per village recruited. Participants aged 6-60 years and 55% were female. | 15% DEET lotion + LLINs | Compliance measured by self-reporting, observed volume of lotion used and random infrequent sniff checks. 58% of participants used lotions >90% of the time. | Placebo lotion + LLINs | Incidence of *P. falciparum* and *P. vivax* infection (confirmed using RDT) | Monthly active detection (5-8 month follow up period, average 6.3 months). |
| Dadzie *et al*. [[2](#_ENREF_2)] | Non-randomized, two village, controlled study | 2010-11 | Ghana | 2 villages (study pop. unclear but 200-350 people recruited for malaria parasite prevalence survey). | NO MAS mosquito repellent (active ingredient not stated) | Self-reported compliance at 3 months = 96% | No repellent | Prevalence of *P. falciparum* infection (confirmed using RDT) | Cross sectional survey at 8 month timepoint. |
| Deressa *et al*. [[3](#_ENREF_3)] | Cluster-randomized controlled trial | 2008 | Ethiopia | 16 rural villages with 1,235 households  3,078 individuals in intervention and 3,004 in control group | Buzz-Off® petroleum jelly and essential  oil blend + LLINs | Not reported | LLINs only | Prevalence of *P. falciparum* and *P. vivax* infection (confirmed using microscopy) | Cross sectional surveys at 1 and 2 month timepoints. |
| Dutta *et al*. [[4](#_ENREF_4)] | Non-randomized cluster allocated factorial trial | 2003-6 | India | Intervention: 306 households  with pop of 1,836; Control: 294 households with a pop. of 1764. | 12% N,N- diethylbenzamide w/w. cream-base tubes (25 mg) (Odomos) | Compliance assessed by unannounced ‘sniff checks’. Figures not reported | No repellent | Malaria (*P. falciparum*) incidence (confirmed using microscopy) | Weekly active case detection (2 year follow up period) |
| Hill *et al*. [[5](#_ENREF_5)] | Double blind, randomized, controlled  trial | 2003 | Bolivia | 4,008 individuals in 860 households in rural villages/peri-urban districts  Participants aged >10 years and 45% were female | *Eucalyptus maculata citriodon* with a PMD concentration of 30% + ITN | Measured by questionnaires, observed volume of lotion used and random sniff checks. 99% of participants used lotions >90% of the time | 0.1% clove oil + ITN | Incidence of *P. falciparum* or *P. vivax* infection - with or without fever (*P. falciparum* confirmed by RDT and *P. vivax* confirmed by blood slide at local clinic) | Monthly active detection for *P. falciparum*.  Passive detection for *P. vivax*.  4 month follow up period. |
| Kroeger *et al*. [[6](#_ENREF_6)] | Matched cluster randomized controlled trial | 1991-2 | Ecuador and Peru | 18 rural communities | Repellent soap containing 20% DEET and 0.5% permethrin | 50-70% when soap was distributed free, 6% when soap was sold | No repellent | Self-reported malaria incidence (period prevalence of attacks)  Parasites species not determined:  manuscript reports that Ecuador = 86% *P. falciparum* and Peru = 100% *P. vivax* | Single survey for recall of malaria attacks in previous 4 months. |
| McGready *et al*. [[7](#_ENREF_7)] | Double blind, individually randomized, placebo controlled trial | 1995-6 | Thailand | 897 women 3-7 mths pregnant recruited from Karen refugee camps in western Thailand | Repellent lotion containing 20% DEET and thanaka (*Limonia acidissima*) | Compliance self-reported at 91% and actively detected at 85% | A placebo formulation containing thanaka | Incidence of *P. falciparum* and *P. vivax* infection ('patent parasitaemic episode' confirmed by microscopy) | Weekly active detection (median follow-up of 18 weeks, range 0-32 weeks) |
| Sangoro *et al*. [[8](#_ENREF_8)] | Cluster-randomized, placebo-controlled trial | 2009-10 | Tanzania | 937 households recruited from a rural village. 50% of households in a village were recruited. Participants were aged >6 mths and 55.3% of household heads were female | 15% DEET lotion + LLINs | Compliance measured by self-reporting and compliance with repellent reported at 89% and placebo 68% | Placebo lotion + LLINs | Malaria (*P. falciparum*) incidence (confirmed using RDT) | Passive case detection (14 month follow-up period) |
| Rowland *et al*. [[9](#_ENREF_9)] | Cluster randomized placebo controlled trial | 1999-2000 | Pakistan | 127 households recruited from a refugee camp on Afghan border. 25% of households in camp were enrolled. Participants were aged >5 yrs and 49.2% were female | 20% DEET and 0.5% permethrin soap | 20 (16%) households interviewed at end of study, 19 (95%) reported using the repellent ‘regularly’ | Placebo lotion | Incidence of *P. falciparum* and *P. vivax* infection (confirmed by microscopy) | Passive detection (5 month follow-up period) |
| Vittal *et al*. [[10](#_ENREF_10)] | Non-randomized, two village controlled study | 1976-1978 | India | 2 rural villages | Insect repellent, proprietary name Enteemosq (active ingredient not stated) | Not estimated | No repellent | Malaria incidence (confirmed by microscopy)  Parasite species not determined: according to expert opinion (Dr. Ramesh Dhiman, National Institute of Malaria Research, India) approx. 85% of cases are *P. vivax* in this area | Active case detection (2 year follow-up period) |

1. Chen-Hussey V, Carneiro I, Keomanila H, Gray R, Bannavong S, Phanalasy S, Lindsay SW: **Can topical insect repellents reduce malaria? A cluster-randomised controlled trial of the insect repellent N,N-diethyl-m-toluamide (DEET) in Lao PDR.** *PloS one* 2013, **8:**e70664.

2. Dadzie S, Boakye D, Asoala V, Koram K, Kiszewski A, Appawu M: **A community-wide study of malaria reduction: evaluating efficacy and user-acceptance of a low-cost repellent in northern Ghana.** *American Journal of Tropical Medicine and Hygiene* 2013, **88:**309-314.

3. Deressa W, Yihdego YY, Kebede Z, Batisso E, Tekalegne A, Dagne GA: **Effect of combining mosquito repellent and insecticide treated net on malaria prevalence in Southern Ethiopia: A cluster-randomised trial.** *Parasites and Vectors* 2014, **7:**1.

4. Dutta P, Khan AM, Khan SA, Borah J, Sharma CK, Mahanta J: **Malaria control in a forest fringe area of Assam, India: a pilot study.** *Transactions of the Royal Society of Tropical Medicine and Hygiene* 2011, **105:**327-332.

5. Hill N, Lenglet A, Arnez AM, Carneiro I: **Plant based insect repellent and insecticide treated bed nets to protect against malaria in areas of early evening biting vectors: double blind randomised placebo controlled clinical trial in the Bolivian Amazon.** *British Medical Journal* 2007, **335:**1023.

6. Kroeger A, Gerhardus A, Kruger G, Mancheno M, Pesse K: **The contribution of repellent soap to malaria control.** *American Journal of Tropical Medicine and Hygiene* 1997, **56:**580-584.

7. McGready R, Simpson JA, Htway M, White NJ, Nosten F, Lindsay SW: **A double-blind randomized therapeutic trial of insect repellents for the prevention of malaria in pregnancy.** *Transactions of the Royal Society of Tropical Medicine and Hygiene* 2001, **95:**137-138.

8. Sangoro O, Turner E, Simfukwe E, Miller JE, Moore SJ: **A cluster-randomized controlled trial to assess the effectiveness of using 15% DEET topical repellent with long-lasting insecticidal nets (LLINs) compared to a placebo lotion on malaria transmission.** *Malaria Journal* 2014, **13:**324.

9. Rowland M, Downey G, Rab A, Freeman T, Mohammad N, Rehman H, Durrani N, Reyburn H, Curtis C, Lines J, Fayaz M: **DEET mosquito repellent provides personal protection against malaria: a household randomized trial in an Afghan refugee camp in Pakistan.** *Tropical Medicine and International Health* 2004, **9:**335-342.

10. Vittal M, Limaye LS: **Field village scale trial of use of repellent in malaria control.** *Indian Journal of Medical Sciences* 1984, **38:**201-203.
