# Supplementary material for: Are topical insect repellents effective against malaria in endemic populations? A systematic review and meta-analysis
Source: Malar J. 2014 Nov 21;13:446. doi: 10.1186/1475-2875-13-446 (PMC4246562; doi:10.1186/1475-2875-13-446)
Supplement: Supplementary file 4 — Additional file 4: Risk of bias assessment. (DOCX 28 KB) [file 12936_2014_3597_MOESM4_ESM.docx]

**Additional File 4: Risk of bias assessment**

| Study | Criteria assessed | | | | | | | | |
| --- | --- | --- | --- | --- | --- | --- | --- | --- | --- |
|  | Sequence generation | Allocation concealment | Baseline outcome measurement similar | Baseline characteristics similar | Loss to follow up | Blinding of outcome assessment | Contamination | Selective outcome reporting | Other bias |
| Chen-Hussey *et al*. [[1](#_ENREF_1)] | Low risk | Low risk | Low risk | Low risk | Low risk | Low risk | Low risk | Low risk | Low risk |
| Dadzie *et al*. [[2](#_ENREF_2)] | Unclear | Unclear | High risk | Unclear | Unclear | Unclear | Low risk | Low risk | High risk* |
| Deressa *et al*. [[3](#_ENREF_3)] | Low risk | Low risk | Low risk | Low risk | Low risk | Low risk | Low risk | Low risk | Low risk |
| Dutta *et al*. [[4](#_ENREF_4)] | Unclear | Unclear | Low risk | Unclear | Unclear | Unclear | Low risk | Low risk | High risk* |
| Hill *et al*. [[5](#_ENREF_5)] | Low risk | Low risk | Low risk | Low risk | Low risk | Low risk | Low risk | Low risk | Low risk |
| Kroeger *et al*. [[6](#_ENREF_6)] | Unclear | Unclear | Low risk | Unclear | Unclear | High risk (self-reported malaria) | Low risk | Low risk | High risk* |
| McGready *et al*. [[7](#_ENREF_7)] | Unclear | Unclear | Unclear | Low risk | Low risk | Low risk | Low risk | Low risk | Low risk |
| Sangoro *et al*. [[8](#_ENREF_8)] | Low risk | Low risk | Unclear | High risk | Low risk | Unclear | Low risk | Low risk | Low risk |
| Rowland *et al*. [[9](#_ENREF_9)] | Low risk | Unclear | Low risk | Low risk | Unclear | Low risk | Low risk | Low risk | Low risk |
| Vittal *et al*. [[10](#_ENREF_10)] | Unclear | Unclear | High risk | Unclear | Unclear | Unclear | Low risk | Low risk | High risk* |
| * Analysis did not take into account clustering | | | | | | | | | |

1. Chen-Hussey V, Carneiro I, Keomanila H, Gray R, Bannavong S, Phanalasy S, Lindsay SW: **Can topical insect repellents reduce malaria? A cluster-randomised controlled trial of the insect repellent N,N-diethyl-m-toluamide (DEET) in Lao PDR.** *PloS one* 2013, **8:**e70664.

2. Dadzie S, Boakye D, Asoala V, Koram K, Kiszewski A, Appawu M: **A community-wide study of malaria reduction: evaluating efficacy and user-acceptance of a low-cost repellent in northern Ghana.** *American Journal of Tropical Medicine and Hygiene* 2013, **88:**309-314.

3. Deressa W, Yihdego YY, Kebede Z, Batisso E, Tekalegne A, Dagne GA: **Effect of combining mosquito repellent and insecticide treated net on malaria prevalence in Southern Ethiopia: A cluster-randomised trial.** *Parasites and Vectors* 2014, **7:**1.

4. Dutta P, Khan AM, Khan SA, Borah J, Sharma CK, Mahanta J: **Malaria control in a forest fringe area of Assam, India: a pilot study.** *Transactions of the Royal Society of Tropical Medicine and Hygiene* 2011, **105:**327-332.

5. Hill N, Lenglet A, Arnez AM, Carneiro I: **Plant based insect repellent and insecticide treated bed nets to protect against malaria in areas of early evening biting vectors: double blind randomised placebo controlled clinical trial in the Bolivian Amazon.** *British Medical Journal* 2007, **335:**1023.

6. Kroeger A, Gerhardus A, Kruger G, Mancheno M, Pesse K: **The contribution of repellent soap to malaria control.** *American Journal of Tropical Medicine and Hygiene* 1997, **56:**580-584.

7. McGready R, Simpson JA, Htway M, White NJ, Nosten F, Lindsay SW: **A double-blind randomized therapeutic trial of insect repellents for the prevention of malaria in pregnancy.** *Transactions of the Royal Society of Tropical Medicine and Hygiene* 2001, **95:**137-138.

8. Sangoro O, Turner E, Simfukwe E, Miller JE, Moore SJ: **A cluster-randomized controlled trial to assess the effectiveness of using 15% DEET topical repellent with long-lasting insecticidal nets (LLINs) compared to a placebo lotion on malaria transmission.** *Malaria Journal* 2014, **13:**324.

9. Rowland M, Downey G, Rab A, Freeman T, Mohammad N, Rehman H, Durrani N, Reyburn H, Curtis C, Lines J, Fayaz M: **DEET mosquito repellent provides personal protection against malaria: a household randomized trial in an Afghan refugee camp in Pakistan.** *Tropical Medicine and International Health* 2004, **9:**335-342.

10. Vittal M, Limaye LS: **Field village scale trial of use of repellent in malaria control.** *Indian Journal of Medical Sciences* 1984, **38:**201-203.
